# Tau Protein Modifications and Interactions: Their Role in Function and Dysfunction

**DOI:** 10.3390/ijms15034671

**Published:** 2014-03-18

**Authors:** Anna Mietelska-Porowska, Urszula Wasik, Marcelina Goras, Anna Filipek, Grazyna Niewiadomska

**Affiliations:** Nencki Institute of Experimental Biology, Polish Academy of Science, 3 Pasteur Street, Warsaw 02-093, Poland; E-Mails: a.mietelska@nencki.gov.pl (A.M.-P.); uwasik@nencki.gov.pl (U.W.); m.goras@nencki.gov.pl (M.G.); a.filipek@nencki.gov.pl (A.F.)

**Keywords:** tau protein, cytoskeleton, microtubule, tau kinases and phosphatases, tau interacting proteins, neurodegenerative disorders, neurotrophic support

## Abstract

Tau protein is abundant in the central nervous system and involved in microtubule assembly and stabilization. It is predominantly associated with axonal microtubules and present at lower level in dendrites where it is engaged in signaling functions. Post-translational modifications of tau and its interaction with several proteins play an important regulatory role in the physiology of tau. As a consequence of abnormal modifications and expression, tau is redistributed from neuronal processes to the soma and forms toxic oligomers or aggregated deposits. The accumulation of tau protein is increasingly recognized as the neuropathological hallmark of a number of dementia disorders known as tauopathies. Dysfunction of tau protein may contribute to collapse of cytoskeleton, thereby causing improper anterograde and retrograde movement of motor proteins and their cargos on microtubules. These disturbances in intraneuronal signaling may compromise synaptic transmission as well as trophic support mechanisms in neurons.

## 1. Introduction

Tau protein belongs to the family of natively unfolded microtubule-associated proteins that binds to microtubules, is involved in their assembly and stabilization [1] and in regulation of the motor-driven axonal transport. Earlier work showed that tau is concentrated predominantly in neuronal axons [2,3]. However, recent data suggest that tau also might play a physiological role in dendrites [4–6]. Six tau isoforms, produced by alternative mRNA splicing of the *MAPT* gene located on chromosome 17q21.31, are expressed in the adult human brain [7]. Each isoform contains either 3 (3R) or 4 (4R) repeat domains responsible for the interaction with microtubules. In the cerebral cortex of healthy adults the amounts of 3R and 4R tau are equal [8]. It has been also found that the expression of tau is roughly two-times higher in grey matter of the neocortex when compared to white matter or to the cerebellum [9].

Tau function depends on its phosphorylation state [10,11]. The incorporation of phosphate groups into tau depends on its conformation and on the balance between the activities of tau kinases and phosphatases. Changes in tau conformation could result in increased phosphorylation and in decreased binding to microtubules which is important in tau-mediated neurodegeneration [12]. Excessively phosphorylated tau accumulates in the somatodendritic compartment of neurons, aggregates and eventually forms neurofibrillary tangles (NFTs) [13]. There is an evidence that soluble overly phosphorylated tau contributes to neuronal dysfunction before its deposition [14]. It has been shown that highly phosphorylated tau interferes with neuronal functions, such as mitochondrial respiration and axonal transport [15,16]. Biochemical and immunostaining data indicate that overphosphorylated, aggregated tau makes up the intracellular filamentous inclusions present in many human neurodegenerative diseases collectively named tauopathies.

Tau excessive phosphorylation and aggregation could be driven by its interaction with several other proteins like β-amyloid, Fyn kinase, Pin1, heat shock cognate Hsc70 and heat shock protein Hsp90, immunophilins FKBP51 and FKBP52, α-synuklein or actin interacting protein PACSIN1. As the consequence of these interactions tau accumulates in dendritic spines, where it suppresses synaptic responses [17,18]. In neurons excessively phosphorylated tau is involved in: microtubule destabilization, impaired axonal transport of substances [19], post-synaptic dysfunction, compromised cell signaling and, as consequence, cognitive impairments ensue [20].

## 2. Tau Protein

Tau protein is widely expressed in the central and peripheral nervous system, but is also present in kidney, lung and testis [21]. Although tau is most abundant in axons [22–25], it is also found in somatodendritic compartments [26] and in oligodendrocytes [27].

Biophysical studies revealed that tau has hydrophilic properties and the protein exists normally as a natively unfolded or intrinsically disordered protein [28,29]. The polypeptide chain of tau is highly flexible and mobile and has only a low content of secondary structures (α-helix, β-strand, poly-proline II helix). Primary sequence analysis demonstrates that the tau molecule contains three major domains, defined on the basis of their microtubule interactions and/or their amino acid character: an acidic *N*-terminal part; a proline-rich region and a basic *C*-terminal domain. Thus, tau protein is a dipole with two domains having the opposite charge [30]. This asymmetry of charges is crucial for interactions between tau and microtubules and other partners as well as for internal folding and aggregation [31].

The *C*-terminal domain binds to microtubules and promotes their assembly and is termed the “assembly domain” [32]. Binding to microtubules occurs through repeated domains (R1–R4) encoded by exons 9–12. Each repeat consists of highly conserved stretches of 18 residues. The repeats are separated from each other by 13- or 14-residue spacer regions [33]. Many studies support a role for the assembly domain in the modulation of the phosphorylation state of tau protein. A direct and competitive binding has been demonstrated between the region of tau containing residues 244–236 (numbering of amino acids is that of the longest human tau) and the microtubule or protein phosphatase 2A (PP2A). As a consequence, microtubules could inhibit PP2A activity by competing for binding to tau [34].

The middle region of tau residues 150–240 contains numerous prolines, which are targets of many proline-directed kinases and binding sites for proteins with SH3 domains. This part of the tau molecule is termed as a “proline-rich domain” [31].

The acidic *N*-terminal part of tau does not bind to microtubules but projects away from the microtubule surface and is termed “projection domain” [35]. This domain of tau may interact with other cytoskeletal elements, mitochondria or neuronal plasma membrane [36–38] and it may determine spacing between microtubules in the axon [39]. In peripheral neurons tau contains an additional *N*-terminal sequence encoded by exon 4A which generates a specific peripheral neuron isoform called “big tau” [40].

## 3. Role of Tau Protein in Neurons

**Figure 1.**
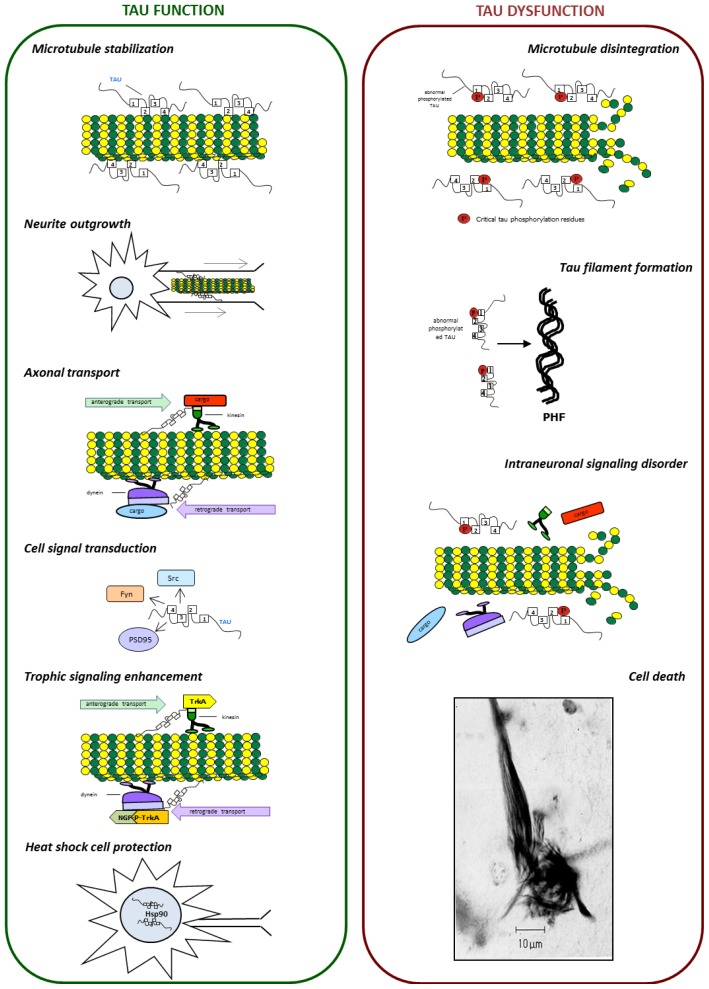
Tau is a multi-functional protein. As a microtubule-associated protein tau contributes to microtubule dynamics and participates in neurite outgrowth, axonal transport and trophic signaling enhancement. Moreover, tau participates in cell signal transduction through the modulation of the activity of Src and Fyn kinases and PSD95 protein. In the nucleolar organizing region of cell, tau can also be involved in DNA repair and heat shock responses (**left panel**). Tau dysfunction leads to microtubule disintegration, tau filaments formation and intraneuronal signaling disorder and, as a consequence, to cell death (**right panel**).

The tau is a multifunctional protein [41–43] (Figure 1). It has numerous binding partners, including signaling molecules, cytoskeletal elements and lipids. The most important function of tau is its role in tubulin polymerization. On tubulin, the tau interacting site is located at the *C*-terminal end, which is highly acidic. Binding of tau to tubulin is regulated by post-translational modifications, especially by phosphorylation. Phosphorylation may neutralize the positive charge [44], alter the conformation and detach tau from microtubules [45]. In pathological conditions, tau self-polymerization and aggregation might also affect the tau-tubulin binding [46]. Tau may interact with microtubules directly and indirectly. Direct interactions include the binding, stabilization and promotion of microtubule assembly [47]. The ability of tau to bind microtubules depends on the microtubule-binding domain and on adjacent regions [48]. Indirect interaction of tau with microtubules affects other proteins that may or may not interact with microtubules by themselves. These interactions may require the projection domain of tau [31,49].

The adult forms of tau promote assembly of microtubules more actively than the foetal form [29]. The recent studies concerning the physical interaction between tau and microtubules indicated that, while all repeats contacted the microtubules, there were specific sequences that were strongly involved in the interaction [50]. These sequences included ^240^KSRLQTAPV^248^, ^275^VQINKKLDLS^285^ and ^297^IKHV^300^. In addition, residues in the flanking regions as far upstream as S214 and as far downstream as L375 were also involved, with ^225^KVAVVRT^231^ and ^370^KIETHK^375^ having especially strong interactions [50,51]. Both sequences ^275^VQINKKLDLS^285^ and ^297^IKHV^300^ are coded by exon 10 and this may explain why 4R tau isoforms interact with microtubules more strongly than 3R tau isoforms [52]. The repeat sequences are thought to directly bind microtubules through their positive net charge, which interacts with negatively charged residues of tubulin monomers [44]. Tau can bind outside and inside of microtubules, with its *N*- and *C*-terminal domains [38,53].

Binding of tau to microtubules can take part in axonal transport and can interfere with the binding of motor proteins [12]. A gradient of tau along the axon with the highest level around the synapse [54] might facilitate the detachment of motor proteins from their cargo near the presynaptic terminal and in consequence might increase axonal transport efficiency [12]. As to the interactions with other cytoskeletal components, tau binds to spectrin and actin filaments [55,56]. This might allow tau-stabilized microtubules to interconnect with neurofilaments that restrict the flexibility of the microtubule lattices [57].

Tau can also act as postsynaptic scaffolding protein. As a scaffold protein, tau modulates the activity of Src tyrosine kinases, c-Src and Fyn, and facilitates c-Src-mediated actin rearrangements [58]. In the case of Fyn, it has been suggested that tau normally tethers Fyn to PSD-95/NMDA receptor signaling complex [4]. In the absence of tau, Fyn can no longer traffic into postsynaptic sites in dendrites. Although normally very little tau is present in dendrites, probably it is enough to ensure proper localization of postsynaptic components [4]. Tau can also act as a scaffold protein in oligodendrocytes, where it might connect Fyn and microtubules in order to enable processes extension [27].

Another function of tau is involvement in growth factor signaling [59,60]. Under NGF stimulation, tau is distributed at ends of cellular extensions, where it associated with actin in a microtubule-independent manner [55]. Tau facilitates signaling through receptors for NGF and EGF, what may increase the activity of the mitogen-activated protein kinase (MAPK). Some data suggest that phosphorylation of tau on threonine 231 is necessary for the growth factor-induced activation of the Ras-MAPK pathway [61]. It remains unverified whether tau interacts directly with growth factor receptors, but it may facilitate signaling by binding to adaptor proteins e.g., Grb2 [62].

## 4. Post-Translational Modifications of Tau

Tau is highly regulated and is subject to a complex array of post-translational modifications. It is modified by serine, threonine and tyrosine phosphorylation [63], isomerization [64], glycation [65], nitration [66], *O*-GlcNAcylation [67], acetylation [68], oxidation [69], polyamination [70], sumoylation [71], ubiquitination [72] and proteolytic cleavage (truncation) [73]. Abnormal post-translational modifications are proposed to be the main cause of the mechanism by which tau protein becomes a non-functional entity. Much of the evidence, to be discussed below, suggests that abnormal phosphorylation is a key event that triggers the pathological aggregation of tau in tauopathies.

### 4.1. Phosphorylation and Dephosphorylation of Tau Protein

Protein phosphorylation is the addition of a phosphate group by esterification at three types of amino acids: serine, threonine and tyrosine. Phosphorylation is the most common tau post-translational modification described. So far, 85 phosphorylation sites have been identified in the tau molecule. The phosphorylation status of the tau is a consequence of the equilibrium between the amount and activity of protein kinases and phosphatases. In neurodegenerative diseases tau undergoes abnormal excessive phosphorylation.

#### 4.1.1. Tau Kinases

Kinases which are involved in tau phosphorylation can be divided into three classes: proline-directed protein kinases (PDPK), non-PDPK protein kinases and tyrosine protein kinases (TPK).

##### 4.1.1.1. GSK-3

Glycogen synthase kinase-3 (GSK-3), belongs to the PDPK class, and is a serine/threonine-specific kinase whose activity is regulated by phosphorylation. GSK-3 is inactivated through phosphorylation of serine 21 (GSK-3α isoform) or serine 9 and 389 (GSK-3β isoform). The activation of GSK-3 depends on the phosphorylation at tyrosine 279 (GSK-3α) or tyrosine 216 (GSK-3β) [74–76]. GSK-3 was identified as a tau protein kinase in the 1990s [77]. So far 42 GSK-3 phosphorylable sites were identified in tau. Among them 29 were phosphorylated in Alzheimer disease (AD) brains [11,78,79]. The level of GSK-3 in tauopathy seems to correlate with the progress of neurodegeneration. The postmortem analysis of brains from AD patients and age-matched control samples indicates that the level of GSK-3 is increased in neurodegeneration [80] and the activity of GSK-3 correlates with the increasing amount of NFTs [81]. Moreover, GSK-3β co-localizes with NFTs [82]. Additionally, studies performed on cultured neurons have shown that the GSK-3 inhibitor, lithium, protects cells against neurodegeneration [83,84]. Abnormally phosphorylated tau protein is also the main component of neurofibrillary tangles found in Parkinson’s disease (PD) [85]. Increased tau phosphorylation at Ser396 by GSK-3β has been discovered in synapse-enriched fractions taken from PD brains [86]. Moreover, tau pathology has been identified in the brains of PD patients with leucine-rich repeat kinase 2 (LRRK2) mutations [87].

DJ-1 is a small protein, the product of a highly conserved gene that has been identified as one of the most frequently mutated genes in familial Parkinson’s disease (PD). Recently, it has been postulated that familial PD-associated DJ-1L166P and DJ-1D149A mutations increase tau phosphorylation by increasing the activity of GSK-3β [88]. The link between tau phosphorylation and GSK-3 has been shown in studies performed on transgenic mice overexpressing mutant human tau (P301L, 4RON). This mutation is related to the tauopathy named frontotemporal dementia with Parkinsonism linked to chromosome 17 (FTDP-17). The treatment with lithium of the aforementioned transgenic mice led to a decrease in the level of tau phosphorylation and the level of aggregated, insoluble tau. A similar result was obtained when another GSK-3 inhibitor, AR-A014418, was used [89]. Moreover, recently the implication of GSK-3 in tau pathology has been confirmed using pR5 mice that express the P301L tau mutation found in familial forms of frontotemporal dementia [90].

##### 4.1.1.2. Cdk5

Cyclin-dependent kinase 5 (cdk5), originally purified as tau kinase II, is a serine/threonine kinase [91] which belongs to the PDPK class. Similarly, as with the case of GSK-3, cdk5 activity is regulated by phosphorylation. Three residues seem to be implicated in this process. Phosphorylation at tyrosine 15 activates cdk5, in contrast to phosphorylation at threonine 14 and at serine 159 that inhibits cdk5 activity [92]. Additionally, the activation of cdk5 requires the binding of an activatory subunit, either p35 or p25 which is generated by calpain-dependent proteolytic cleavage of p35 [92]. The cdk5 activator, p25, has a long half-life and is involved in aberrant cdk5 activity toward tau [93,94]. It has been shown that p25 accumulates in the tauopathic brains derived from AD patients. The p25/cdk5 holoenzyme phosphorylates tau and reduces its ability to bind to microtubules. Studies performed on primary neurons have shown that p25/cdk5 alters cells morphology, causes cytoskeletal disruption and apoptotic cell death [95]. Silencing of cdk5 reduces the phosphorylation of tau in primary neuronal cultures and in the brain of wild type C57BL/6 mice. In a triple transgenic mouse model of AD disease, the knock-down of cdk5 strongly decreases the number of neurofibrillary tangles in the hippocampus [96]. Cdk5 also seems to be involved in the regulation of tau phosphorylation by GSK-3. Tau, cdk5, and GSK-3 are components of a 450-kDa complex in which cdk5 phosphorylates tau and primes it for phosphorylation by GSK-3 [97]. Recently, it has been shown that the effect of cdk5 on tau phosphorylation depends on the Pin 1 protein [98–100].

##### 4.1.1.3. JNK

C-Jun amino-terminal kinase (JNK) belongs to the PDPK group of kinases and simultaneously to the family of serine and threonine mitogen-activated protein kinases (MAPKs). JNK phosphorylates tau at 12 sites, which were identified only in neurodegeneration and not in control conditions. Immunohistochemical analysis of JNK expression in AD, Pick’s disease (PiD), progressive supranuclear palsy (PSP) and corticobasal degeneration (CBD) unveiled its co-localization with tau aggregates [101]. Moreover the increased level of JNK has been observed in AD brains and its activated form (p-JNK) co-localizes with p-tau in neurons of AD patients [102,103].

##### 4.1.1.4. CK1

Casein kinase 1 (CK1) is a family of protein kinases, non-PDPK, which in humans consists of six isoforms derived from distinct genes with further diversity generated by alternative splicing [104]. CK1 can phosphorylate tau at Ser202/Thr205 and Ser396/Ser404 *in vitro* and in cell culture, modulating its binding affinity for microtubules [105–107]. The distribution of CK1 delta (CK1δ) was studied by immunohistochemistry. CK1δ co-localizes with NFTs in AD, Down syndrome (DS), PSP, parkinsonism dementia complex of Guam (PDC) and with Pick bodies in PiD [108]. Moreover, the mRNA of CK1δ is upregulated in brain derived from AD patients. There was a 24.4-fold increase in CK1δ mRNA in hippocampus, 8.04-fold in the amygdala, 7.45 in the entorhinal cortex and 7.30-fold in the mediotemporal gyrus of AD when compared to control brains [109].

##### 4.1.1.5. Dyrk1A

Increased non-PDPK, Dyrk1A (Dual specificity tyrosine-phosphorylation-regulated kinase 1A) kinase immunoreactivity has been found in the cytoplasm and nuclei of scattered neurons of the neocortex, entorhinal cortex, and hippocampus in AD, DS, and PiD [110]. Dyrk1A protein phosphorylates the microtubule-associated protein tau at several sites, including Thr181, Ser199, Ser202, Thr205, Thr212, Thr217, Thr231, Ser396, Ser400, Ser404, and Ser422. Phosphorylation by Dyrk1A primes further phosphorylation of tau by GSK-3 at Thr181, Ser199, Ser202, Thr205, and Ser208 but not by cdk5 and PKA [111–113]. Tau phosphorylation at Thr212, Ser202 and Ser404 is the hallmark of AD and is significantly increased in Dyrk1A transgenic mice overexpressing human Dyrk1A [110]. Moreover, a study performed using a transgenic mouse model of DS, the Ts65Dn mice, confirmed the abnormal phosphorylation of tau upon increased Dyrk1A activity. Dyrk1A induced tau phosphorylation inhibited tau activity to stimulate microtubule assembly and promoted its self-assembly into filaments [109,110].

##### 4.1.1.6. AMPK

Adenosine-monophosphate activated protein kinase (AMPK), non-PDPK, is a heterotrimeric serine/threonine kinase. The phosphorylation of tau by AMPK takes place at several residues and effects tau binding to microtubules [114,115]. *In vitro* assays showed that AMPK can directly phosphorylate tau at Thr231 and Ser396/404. Activated/phosphorylated AMPK (p-AMPK) was abnormally accumulated in cerebral neurons in tauopathies such as AD, tangle-predominant dementia, PDC, PiD, and FTDP-17. Granular p-AMPK immunoreactivity was observed in apparently unaffected neurons devoid of tau inclusion, suggesting that AMPK activation preceded tau accumulation. Phospho-AMPK was not found in purified PHFs, indicating that p-AMPK did not co-aggregate with tau in tangles [114].

##### 4.1.1.7. MARKs

The microtubule-affinity regulating kinases (MARKs) belong to the AMPK branch of the CAMK (calcium/calmodulin-dependent protein kinase) group of kinases [116]. MARKs belong to the non-PDPK group of kinases. The MARK protein family consists of four highly conserved members (MARK1–4). MARK kinases co-localize with NFTs, and the expression level of MARK proteins have been shown to be elevated in AD brains [117]. MARKs phosphorylate tau protein at the KXGS motif of its repeat domains. This phosphorylation leads to the detachment of tau protein from microtubules and in consequence, to destabilization of the cytoskeleton and the tau aggregation [118,119]. Tau phosphorylation by MARKs occurs at Ser262, 293, 324 and 356 [120,121]. Recently, it has been postulated that MARK4 is the crucial isoform of the MARK family which is implicated in the pathological phosphorylation of tau [122]. Studies concerning the regulation of MARKs activity have shown that MARK1 and MARK2 are activated by DAPK (death-associated protein kinase) and DAPK^−^/^−^ mice brain displays a reduced phosphorylation of tau [123].

##### 4.1.1.8. PKA

Cyclic AMP (cAMP)-dependent protein kinase (PKA) is a serine/threonine protein kinase which belongs to the non-PDPK class. PKA catalyzes tau phosphorylation *in vitro* and *in vivo* [124–126]. The phosphorylation of tau protein by PKA triggers subsequent tau phosphorylation by GSK-3β at several AD-relevant phosphorylation sites (Thr181, Ser199, Ser202, Thr205, Thr217, Thr231, Ser396 and Ser422) and simultaneously inhibits tau phosphorylation at Thr212 and Ser404. Additionally, prephosphorylation of tau by PKA slightly promotes tau phosphorylation by cdk5 kinase at Ser396 and inhibits its phosphorylation at Ser202, Thr212, Thr217 and Ser404 [123]. Abnormal phosphorylation of the tau protein by PKA kinase was confirmed *in vivo* [127]. It has been also observed that infusion of PKA activator, forskolin, into the lateral ventricle of brain in adult rats induced activation of PKA by several fold and concurrently enhanced the phosphorylation of tau.

##### 4.1.1.9. TPKI and TPKII

Tau protein kinase I (TPKI) is a non-PDPK that can phosphorylate native tau isolated from normal brain, which is already phosphorylated to some extent, but it can not phosphorylate completely dephosphorylated tau. In contrast, TPKII can phosphorylate also the latter form of tau [128]. The tau residues phosphorylated by TPKII were Ser202, Thr205, Ser235, and Ser404, while those by TPKI were Ser199, Thr231, Ser396, and Ser413 [129]. Interestingly, the TPKII-dependent tau phosphorylation increased with increasing Aβ concentration [130]. Similar interaction has been postulated in the case of TPKI [131].

#### 4.1.2. Tau Phosphatases

Protein phosphatases (PPs), responsible for dephosphorylation of tau include: PP2B, PP2A, PP1 and PP5 [132].

##### 4.1.2.1. PP2B

Protein phosphatase PP2B (calcineurin) is one of the major serine/threonine phosphatases in the brain the activity of which depends on Ca^2+^/calmodulin. It consists of a catalytic A subunit with molecular mass of about 63 kDa and a regulatory B subunit with molecular mass of about of 19 kDa, which binds Ca^2+^ and shares some degree of homology with calmodulin. The results obtained using phosphorylation-sensitive monoclonal antibodies AT-180 (against tau phosphorylated on Thr231) and AT-270 (for Thr181) show that reduction of PP2B activity in brain by antisense oligonucleotides led to persistent phosphorylation of tau at Thr181 and Thr231 [133]. Studies performed by Rahman *et al.* [134] showed that PP2B purified from AD brains efficiently dephosphorylated p-tau. The authors found also that the purified PP2B dephosphorylated tau obtained from AD brain at Ser199, Thr217, Ser262, Ser396 and Ser422 with the preferential dephosphorylation at Ser262 and Ser396. Interestingly, no significant difference in PP2B activity was found between control and AD brain in contrast to the results obtained by Qian *et al.* [135] who showed a 3-fold increase in PP2B activity in AD brain as compared to control one. The study of Kim *et al.* [136] concerning the PP2B phosphatase revealed that it can catalyze dephosphorylation of the Ser9 residue on GSK-3β. The overexpression of a constitutively active PP2B mutant (A beta 1–401) increased GSK-3β activity and in consequence phosphorylation of tau. Thus, PP2B similarly to other phosphatases might also act indirectly on tau phosphorylation.

##### 4.1.2.2. PP2A

Protein phosphatase 2A (PP2A) is a major brain tau phosphatase *in vivo* and thus its reduced activity might be a factor contributing to increased tau phosphorylation [137]. PP2A contains a catalytic C subunit, a scaffold-like A subunit and a regulatory PR55/Bα (PP2A_T55α_) subunit. By using the NMR spectroscopy, Landrieu *et al.* [138] determined the dephosphorylation rates of p-tau by PP2A and showed kinetic data for the individual sites including Ser202/Thr205 and Thr231. The authors demonstrated the importance of the PR55/Bα regulatory subunit of PP2A in this enzymatic process, and showed that phosphorylation at the tau Thr231 site inhibits dephosphorylation of the tau Ser202/Thr205 sites. This effect could be released by the Pin1 isomerase. Because this Pin1 effect is lost with the dimeric PP2A core enzyme (PP2A_D_) or when using a tau mutant, Thr231A that cannot be phosphorylated at residue 231, the authors proposed that Pin1 regulates the interaction between the PR55/Bα subunit and the Thr231 epitope on tau. Protein phosphatase PP2A also dephosphorylates tau protein at Ser202/Thr205 in response to microtubule depolymerization [139]. Sontag *et al.* [140] reported that microtubule associated protein 2 (MAP2) is dephosphorylated by endogenous PP2A/Bα (a major PP2A holoenzyme containing PR55/Bα regulatory subunit), in the gray matter of bovine brain. By applying *in vitro* binding assays, the authors showed that PP2A/Bα binds to MAP2c isoforms through a region encompassing the microtubule-binding domain and upstream proline-rich region. The protein-tyrosine kinase Fyn binds to the proline-rich RTPPKSP motif conserved in both MAP2 and tau and inhibits the interaction of PP2A/Bα with either tau or MAP2c. This points to a critical role of Fyn-binding motif in MAP2 and tau in regulating signaling enzymes like PP2A/Bα and Fyn. Dysfunction of these protein complexes is likely to contribute to tau deregulation, microtubule disruption, and altered signaling in tauopathies. All these data, together with the observation that PP2A is normally bound to microtubules in intact cells, suggest that the polymerization state of microtubules could modulate the phosphorylation state of tau at specific sites in normal and AD brain. Thus one can suggest that PP2A and its regulatory subunits might be a therapeutic target for Alzheimer’s disease. It should be also mentioned that modulation of PP2A activity in AD brain might be due to its interaction with an inhibitor called SET/inhibitor 2 (I2) or ARPP-19 [141–143].

##### 4.1.2.3. PP1

Protein phosphatase 1 (PP1) plays a fundamental role in many calcium-dependent cellular processes in neurons. Its catalytic subunit interacts with as many as 200 distinct regulatory proteins that target PP1 to specific subcellular locations where they influence its substrate specificity [144]. PP1 requires metal ions and its maximal activation is seen in the presence of Mn^2+^. Dephosphorylation of excessively phosphorylated tau obtained from AD brain by PP1 seems to be site-specific since PP1 preferentially dephosphorylated Thr212 (40%), Thr217 (26%), Ser262 (33%), Ser396 (42%) and Ser422 (31%) [145]. Residue Thr212 was suggested to be dephosphorylated by PP1 only and not by PP2A or PP2B. This observation, although interesting, has not yet been confirmed. In other recent studies, protein phosphatase PP1 and tau have been linked to deficits in axonal transport [146,147].

##### 4.1.2.4. PP5

Protein phosphatase 5 (PP5) is a phosphatase ubiquitously present in different mammalian tissues including brain, where it is abundantly expressed. Up to now, few physiological substrates of this phosphatase have been identified. Studies performed by Liu *et al.* [148] showed that dephosphorylation of p-tau by PP5 had a similar *Km* to that found for phosphatase PP2A and was within the range of intraneuronal tau concentration. Phosphatase PP5 dephosphorylates tau at all 12 AD-associated abnormal phosphorylation sites studied, with different efficiency toward each site. The most favorable sites for action of PP5 on tau are Thr205, Thr212, and Ser409, less favorable sites being Ser199, Ser202, Ser214, Ser396 and Ser404 and the poorest site is Ser262. The activity but not the amount of PP5 was found to be decreased by about 20% in AD neocortex which suggests that the attenuated activity of this phosphatase might be responsible for the overphosphorylation of tau in this disease. Recently, it has been shown that PP5 binds calcium binding proteins: S100A1, S100A2, S100A6 or S100B and that these S100 proteins activate PP5, when checked using tau as a physiological substrate [149]. The association of PP5 with S100 suggests a Ca^2+^-dependent mechanism of tau dephosphorylation. It is of note that the level of Ca^2+^ and of calcium binding proteins in most neurodegenerative diseases, including AD, is deregulated [150].

##### 4.1.2.5. CacyBP/SIP

Recent study [151] suggests that the calcyclin binding protein and Siah-1 interacting protein (CacyBP/SIP) protein, dephosphorylates tau. Similar to PP5, CacyBP/SIP phosphatase activity toward tau is affected by a calcium-binding protein, S100A6. The observed inhibition of CacyBP/SIP tau phosphatase activity might be a result of the influence of S100A6 on the CacyBP/SIP phosphorylation state. CacyBP/SIP is expressed in different tissues with the highest level being found in the brain. It is mainly a neuronal protein interacting with different targets. Among them are tubulin, actin and tropomyosin, which suggest that CacyBP/SIP might play a role in cytoskeletal reorganization. Furthermore, dephosphorylation of tau protein [151] and of ERK1/2 kinase [152] by CacyBP/SIP indicate that this phosphatase might play a role in signaling pathways leading to cell proliferation and differentiation. In our study [151], we have also found that in AD patients and line 1 tau transgenic mice, changes in cellular distribution of CacyBP/SIP were similar to those observed for two other microtubule proteins, β-tubulin and tau.

##### 4.1.2.6. TNAP

Tau protein released on death of neurons may also induce a neurotoxic effect on hippocampal neurons by activation of the M1 and M3 muscarinic receptors. An essential component that links both effects is a tissue-nonspecific alkaline phosphatase (TNAP) [153]. TNAP is abundant in the central nervous system and is mainly required to keep control over the extracellular levels of phosphorylated compounds. TNAP dephosphorylates overphosphorylated tau once it is released upon neuronal death. Only the dephosphorylated tau behaves as an agonist of muscarinic M1 and M3 receptors, provoking a robust and sustained intracellular calcium increase finally triggering neuronal death. An increase in TNAP activity together with increase of protein and its transcript level were detected in AD patients. These observations indicate that TNAP promotes the neurotoxicity of extracellular tau which contributes to the spread of pathology in AD.

### 4.2. Other Post-Translational Modifications

The state of tau phosphorylation could be influenced by other post-translational modification. The temporal sequence of glycosylation, glycation, nitration, oxidation, polyamination, sumoylation and ubiquitination is unclear, but these modifications seem to occur before tau excessive phosphorylation and NFTs formation [154].

Glycosylation is the covalent attachment of oligosaccharides to a protein. There are two types of glycosylation: *N*-glycosylation and *O*-glycosylation. *N*- and *O*-glycosylation result from the attachment of a sugar on the amine radical of asparagine on the hydroxyl radical of serine or threonine, respectively. Protein tau can be *O*-GlcNAc-ylated (*O*-glycosylation achieved by the engraftment of *N*-acetyl-glucosamine) *in vitro* in recombinant systems and in some transfected cell-lines in culture [155]. *In vivo*, *O*-GlcNAc-ylation has been shown to reduce tau phosphorylation in rat cortex and hippocampus [156]. Conversely, biochemical evidence for tau to become *O*-GlcNAc-ylated was not obtained in the study of Borghgraef *et al.* [157] in Tau. P301L mice chronically treated with Thiamet-G, β-*N*-acetyl-glucosaminidase inhibitor. In AD patients, a negative correlation has been reported between *O*-GlcNAcylation level and tau phosphorylation, suggesting that *O*-glycosylation of tau negatively regulates its phosphorylation [158]. Tau proteins from brains of AD patients and not that from brains of control patients were found to be non-physiologically glycosylated. On the basis of findings described by Takahashi *et al.* [159] the first step in the cascade of events leading to final tau modification is a down-regulation of tau glycosylation which cause the conformational changes leading to exposure of sites for their phosphorylation. The down-regulation of glycosylation and over-activation of GSK3β, in turn facilitates abnormal tau phosphorylation.

Some evidence suggests that tau glycation prevents tau degradation and promotes its accumulation [160]. Moreover, glycation triggers the production of free radicals amplifying oxidative stress, which in turn increases tau phosphorylation [161]. By this mechanism, tau can be oxidized at C322, leading to PHF assembly [69]. Furthermore, oxidative stress promotes tau nitration which indicates that tau glycation can indirectly induce both tau oxidation and nitration, leading to tau phosphorylation and oligomerization [162]. Polyamination promotes NFT formation [163] and, together with tau glycation and nitration may render abnormally phosphorylated tau less prone to biochemical degradation by ubiquitin/proteasome system [164,165]. Subsequently, tau sumoylation can counteract ubiquitination and thus promotes tau aggregation. In this way sumoylation may control level of aggregates of tau [166].

## 5. Proteins Interacting with Tau

As described above, tau protein has several functions in the nerve cells. These functions are supported by a large number of other proteins, which include proteins that affect the phosphorylation of tau, and other proteins relevant to this modification. These proteins are discussed below.

### 5.1. Amyloid-β

Apart from showing nerve and synapse loss, the brains of patients with AD are characterized not only by neurofibrillary tangles NFTs but also by amyloid-β (Aβ)-containing plaques. Aβ is a group of peptides that are structurally homologous, but with different chain length, containing 39–42 amino acids. Aβ is processed from a larger amyloid precursor protein (APP) [167].

Results from both cellular and transgenic animal models indicate that tau protein is essential for Aβ-induced neurotoxicity [168]. In the early 90s the “amyloid cascade hypothesis” was presented. It was postulated that formation of neuritic plaques would stimulate subsequent pathological events, including the formation of NFTs and disruption of synaptic connections, which would lead to reduction in neurotransmission, death of tangle-bearing neurons and dementia [169].

Although Aβ and tau protein become toxic through the different mechanisms, human, animal and *in vitro* studies have found a direct link between Aβ and tau in causing toxicity in AD. Ittner and Götz [170] suggested three possible ways of interaction between both proteins: (1) Aβ drives tau pathology; (2) synergistic toxic effects of Aβ and tau; and (3) tau may mediate Aβ toxicity. The same authors put forward the “tau axis hypothesis” which implies that the converging point of the pathological effects of both proteins is a dendritic area of nerve cells. The hypothesis suggests that increased concentrations of tau within the dendrites can make neurons more vulnerable to damage caused by Aβ in the postsynaptic dendrites [170].

There are strong experimental data indicating that tau is essential for Aβ-induced neurotoxicity. For example, cultured hippocampal neurons from tau knock-out mice are protected against Aβ pathology. The tau silencing in cultured hippocampal neurons from wild-type mice showed that tau was required for pre-fibrillar Aβ-induced microtubule disassembly [168]. Also, reduction of tau prevents Aβ-induced defects in axonal transport of mitochondria [171]. Aβ and pathological P-tau co-localize in AD synapses [172,173]. Other studies revealed that Aβ and/or chronic oxidative stress are critical for development of tau pathology, including tau excessive phosphorylation and NFT formation [161,174].

### 5.2. Pin1

Pin1 is a peptidyl-prolyl isomerase that recognizes a specific motif of a phosphorylated serine or threonine residue preceding a proline residue. Pin1 was first described as a nuclear protein which can regulate a subset of mitotic and nuclear substrates, but its function is not restricted to cell cycle control but is extended to multiple cellular processes such as transcription and apoptosis. Pin1 was shown to be involved in tauopathies since Pin1 dysfunction may have critical consequences on tau pathological aggregation and neuronal death [175]. Recent study performed by Kimura *et al.* [100] shows that Pin1 stimulates dephosphorylation of tau phosphorylated by cdk5. Pin1 binds to tau and stimulates its dephosphorylation at all cdk5 phosphorylation sites including Ser-202, Thr-205, Ser-235, and Ser-404. Tau carrying the FTDP-17 mutation, P301L or R406W, showed slightly weaker binding to Pin1 than wild type tau, suggesting that FTDP-17 mutations induce cdk5-dependent increased tau phosphorylation by reducing its interaction with Pin1. These results demonstrate that mutation of tau may change the conformation of tau, thereby suppressing dephosphorylation and potentially contributing to the etiology of tauopathies [99,176].

Exposure of neurons to Aβ results in dephosphorylation of Pin1, its activation and dephosphorylation of tau on Thr231. This effect might be prevented by Pin1 inhibitor or by okadaic acid which inhibits PP2A [177]. Also, it was found that Pin1 is responsible for the transient modulation of tau phosphorylation at Ser199, Ser396, Ser400 and Ser404 in response to Aβ [178]. Some other data suggest that Pin1 is also involved in the regulation of APP processing and Aβ production [11]. Phosphorylation of Thr743 in APP allows Pin1 to bind to APP [179]. Thus, the observed loss in Pin1 in advanced AD is in agreement with the reported effects of Pin1 in cellular and animal models.

### 5.3. Fyn Kinase

Fyn is a membrane-anchored non-receptor tyrosine kinase from the Src-family. Recent evidence indicates the importance of tau interactions with Fyn during Aβ-mediated neurodegeneration [4,180,181]. Tau phosphorylated by Fyn on Tyr18 [182,183] has been detected in the proportion of tangles in early AD brain [63,184]. Tau interacts with Fyn by its SH3 domain [62]. Binding of tau to SH3 domain is regulated by phosphorylation of tau on specific serine/threonine residues [185].

Tau binds to Fyn in dendritic spines, and this interaction regulates *N*-methyl-d-aspartate (NMDA) receptor signaling [4]. Pathological tau may participate in localization of Fyn in the postsynaptic compartment, where it phosphorylates NMDA receptor subunits which leads to an increase in Ca^2+^ and to excitotoxicity [186]. Ittner *et al.* [4] have suggested that interaction between tau and Fyn in dendrites plays a critical role in mediating Aβ-induced neurotoxicity by influencing the stability of complexes formed by NMDA receptor and postsynaptic density protein 95 (PSD-95). It is conceivable that tau and Fyn might exist in a complex with NMDA receptors and PSD-95 in neurons. Activation of signaling pathways that lead to increased activity of Fyn could therefore affect the tyrosine phosphorylation of tau, which could potentially modulate complex formation, and result in altered trafficking into neuronal membrane compartments. Additionally, Fyn-tau interaction plays an important role in oligodendrocytes, where it regulates the outgrowth of cytoplasmic processes on the glial cell body. Impairment of the tau-Fyn interaction and excessive phosphorylation of tau leads to hypomyelination of axons [27]. All these findings suggest that tau-Fyn interaction is important for tau localization in neurons and has significant implications during the progression of neurodegenerative diseases.

### 5.4. Heat Shock Proteins

Heat shock proteins, called also molecular chaperones, are highly conserved proteins. They are involved in most aspects of protein synthesis, folding, trafficking and assembly of multiprotein complexes [187,188].

The emergence of molecular chaperones as key regulators of tau processing suggests that conformational changes of this protein may be important events in the pathogenesis of AD and other tauopathies. In a cellular environment, post-translational processing of tau is regulated by the chaperone network [189].

The Hsp70 family consists of 13 proteins, some of them were first described as regulators of tau. The most abundant proteins present in the cytoplasm are the constitutive heat shock cognate 70 protein (Hsc70) and the inducible heat shock protein Hsp70. These two proteins share 92% homology in the amino acid sequence. Both have highly conserved *N*-terminal ATPase domains and substrate-binding domains situated just above more variable/regulatory domain [190]. Hsp70 has a dual role with tau. It stabilizes binding of tau to microtubules as well as promoting tau degradation in combination with chaperone-associated ubiquitin ligase (CHIP) [189]. Hsc70 and Hsp70 bind tau, but in the cytosol, endogenous Hsc70 is more abundant than Hsp70 [190]. Recent study has demonstrated that Hsc70 regulates the association of tau with microtubules [191]. The authors found that Hsc70 facilitates MC1 conformation, which is an epitope created when the amino acids at residues 7–9 interact with residues 312–342. They speculated that tau folding into the MC1 conformation after microtubule destabilization could be a protective mechanism to control the disordered nature of tau and prevent self-assembly in neuron. They also found that Hsc70 enhances tau-mediated microtubule polymerization. The work from Miyata *et al.* [188] has shown that inhibition of the ATPase activity of Hsp70/Hsc70 promotes proteasomal degradation of tau while its activation results in tau accumulation. Furthermore, such inhibition was able to reduce p-tau levels and improve cognition in a transgenic mouse model.

Heat shock protein 90 (Hsp90) was also described as a tau-binding protein [192]. It has been shown that Hsp90 promotes tau phosphorylation by its ability to regulate GSK-3β. These data may suggest that Hsp90 allows accumulation of highly phosphorylated tau species. Additionally, other groups report that inhibition of Hsp90 by 17-AAG and other inhibitors reduces cellular levels of two p-tau species, p-Tau(Ser-202/Thr-205) and p-Tau(Ser-396/Ser-404) both of which are important for AD pathogenesis [193]. Dickey *et al.* [194] showed that protein kinase Akt and ubiquitin ligase CHIP co-regulate tau degradation through coordinated interactions involving Hsp90. They suggest that, by regulation of the CHIP/Hsp90 complex, Akt reduced tau ubiquitination and slowed its degradation. In addition, Akt enhances phosphorylation of tau at Ser262/Ser356, a site that is not recognized by the CHIP/Hsp90 degradation complex.

### 5.5. FKBP51 and FKBP52 Immunophilins

In general, immunophilins are cytoplasmic proteins and their physiological function is that of a chaperone with peptidyl-prolyl *cis-*/*trans-*isomerase (PPIase) activity. FKBP51 and FKBP52 are both involved in tau protein turnover [195]. FKBP51 overexpression preserves tau in cells and protects it from ubiquitination, perhaps by twisting tau in such a way as to prevent access to ubiquitin ligases. It was also proposed that phosphorylation of tau drives the association of FKBP51 with tau, suggesting that as tau dissociates from the microtubules, it is recognized by the chaperone machinery and primed for dephosphorylation. FKBP51 promotes the association of tau with Hsp90 which leads to its dephosphorylation [192] and its overexpression enhanced neuronal loss in the rTg4510 tau transgenic mouse model [196]. FKBP51 can work with Hsp90 to produce oligomeric tau in the brain and prevent tau clearance thus increasing tau toxicity. This activity of Hsp90 in cooperation with FKBP51 is in contrast to the effects of other chaperones that have been shown to enhance tau clearance, block amyloid formation, and decrease tangle load in the brain.

Chambraud *et al.* [197] reported that FKBP52, which is abundant in brain, binds directly and specifically to tau, especially to its highly phosphorylated form. Both proteins co-localize in the distal part of the axons of cortical neurons where FKBP52 decreases tau ability to promote microtubule assembly. Furthermore, overexpression of FKBP52 in differentiated PC12 cells prevented the accumulation of tau and resulted in reduced neurite length.

### 5.6. α-Synuclein

α-Synuclein (α-SN) has been found in the Lewy body inclusions that are pathognomic for Parkinson’s disease (PD). It has been suggested that α-SN may be involved in pathogenesis of AD [198] based upon the fact that it binds to tau and primes it for action of kinases. α-Synuclein is abundant in the brain and interacts with synaptic vesicles at presynaptic terminals. There is also evidence for its chaperoning action for other proteins. In biochemical properties α-synuclein resembles tau protein in several respects: it is an acidic, heat-stable, unfolded protein that has characteristic repeats. It aggregates when it is overexpressed [199]. Recent studies show that α-synuclein has the ability to stimulate tau phosphorylation by GSK-3β through formation of a protein complex with these two proteins. The expression of α-SN is promoted by oxidative stress. Accumulation of α-SN induced by such stress may lead to the excessive phosphorylation of tau by GSK-3β [200].

The studies concerning the physiological correlation between tau and α-synuclein have also demonstrated that phosphorylated tau is present in Lewy bodies, which are cytoplasmic inclusions formed by abnormal aggregation of α-SN. The PD-linked neurotoxin 1-methyl-4-phenyl-1,2,3,6-tetrahydropyridine (MPTP) increases the phosphorylation of tau as well as the protein level of α-SN in cultured neuronal cells, and also in mice [200]. Other studies [201] have shown, that α-SN interacts directly with tau and stimulates its phosphorylation by protein kinase A (PKA). PHF-tau is phosphorylated on at least 21 sites. One of these sites, Ser262 is uniquely located within the first microtubule-binding region of tau. Phosphorylation at this site alone was found to detach tau from microtubules, cause microtubule instability, and make tau neurotoxic in *Drosophila* and in cultured primary neurons [202]. PKA phosphorylates tau at both Ser214 and Ser262. It has been discovered that in the presence of α-synuclein, PKA phosphorylates Ser262 to a higher extent than in its absence. These results indicate that α-synuclein is a regulator of phosphorylation of tau at Ser262. Phosphorylation of tau at Ser262 depends also on pathogenic mutations in α-synuclein.

### 5.7. PACSIN1

PACSIN1 (or SYNDAPIN1) is a neuron-specific member of the cytoplasmic adapter proteins PACSINs family. All PACSINs represent a group of the larger Pombe Cdc15 homology (PCH) protein family, which participate in rearrangements of actin networks during vesicle formation and transport [203]. In their study Grimm-Günter *et al.* [204] have shown that PACSINs contribute to tubulin nucleation and retard microtubule regrowth. They also suggested that other PCH proteins have been linked with microtubule and/or centrosome function, mainly by their *N*-terminal F-BAR domains. Additionally, reduction of these proteins levels delay, but do not prevent, tubulin polymerization. Neuron-specific PACSIN1 contains a highly conserved SH3 and F-BAR domain, sequence determining PACSIN1 involvement in F-actin cytoskeleton organization and membrane trafficking [205]. PACSIN1 interacts with vesicle-associated proteins, including large GTPase DYNAMIN1 and EHD proteins, and it plays an important role in endocytosis and endosomal recycling.

PACSIN1 was also identified by Liu *et al.* [205] as a tau-binding protein. PACSIN1-tau interaction reduces tau affinity to microtubules and suppresses tau-induced microtubule polymerization, stability and bundling. These authors used a model system of cultured dorsal root ganglia (DRG) neurons and found that PACSIN1 blocking resulted in a higher number of straight and spread microtubules and decreased axonal length with a greater number of primary axonal branches.

## 6. Tau Dysfunction

### 6.1. Tau Aggregates

Tau aggregates display different morphologies in different tauopathies. The type of aggregate formed is determined by the tau isoforms involved and the presence of mutations in the tau gene [206]. In neurodegenerative diseases, such as AD and AD-related tauopathies (foldopathies) [207], tau is highly phosphorylated and has a tighter more folded conformation and is remarkably more susceptible to aggregate than non-phosphorylated tau [208,209]. The increased pool of soluble tau undergoes additional conformational changes, which may support initial steps of tau assembly into filaments [210]. Much evidence confirms that abnormal phosphorylation converts tau from a biologically functional molecule into a toxic protein, and that this is responsible for the polymerization of tau into paired helical filaments (PHFs), pathological structures observed in AD [211,212]. The PHFs in turn bundle into neurofibrillary tangles or neuropil threads leading to neuronal death. Neurons accumulate misfolded protein deposits recognized by antibodies against tau of 55 to 69 kDa and ubiquitin, and this is accompanied by PHF formation and tubulin fragmentation and deacetylation [208,213]. The deposits tend to fill the basal pole of pyramidal neurons, encompassing the area of the axon hillock and basal dendritic branches.

**Figure 2.**
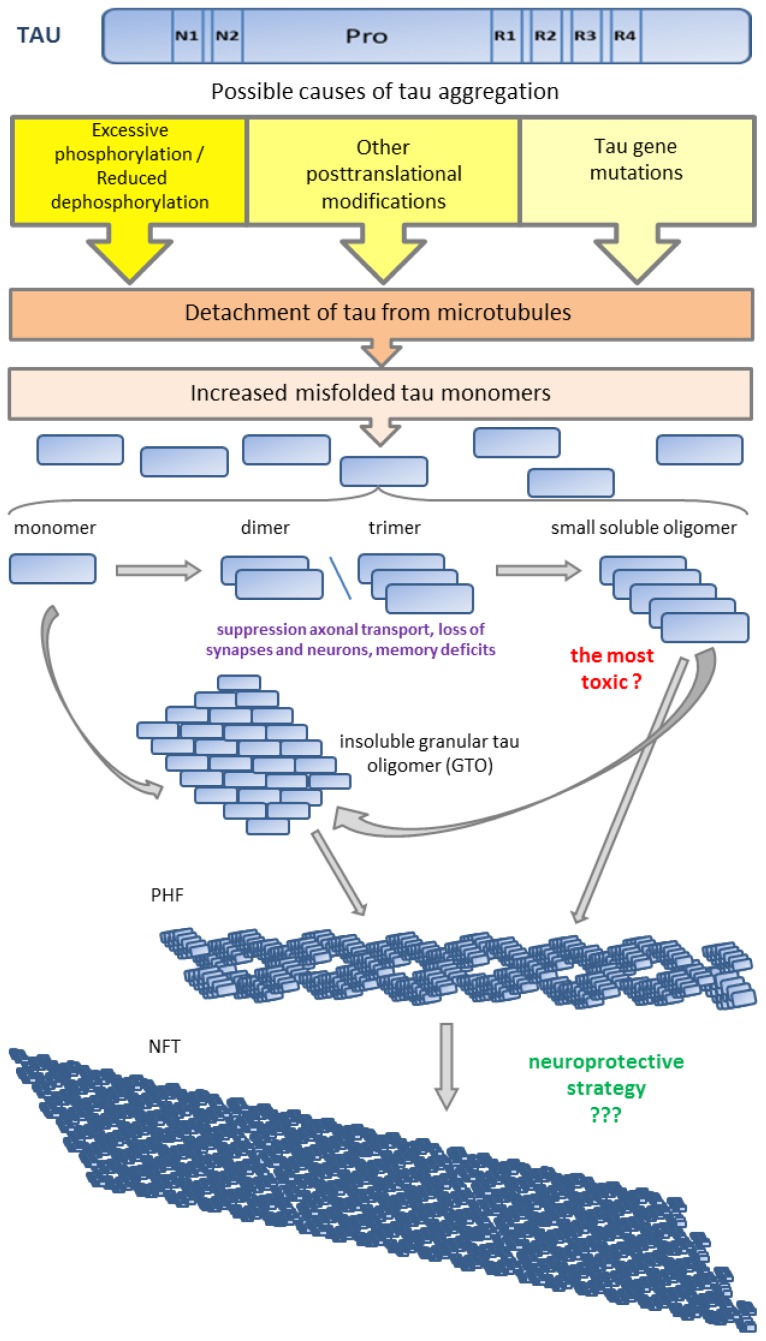
Proposed sequence of stages leading to tau pathology. Detachment of tau from microtubules increases amount of misfolded tau monomers. Monomers aggregate into small soluble tau oligomers. Small soluble tau oligomers and tau monomers can proceed to form granular tau oligomers (GTOs). Probably both, small oligomers and GTOs form paired helical filaments (PHFs) but GTOs are considered to be the main precursors of PHFs. Subsequently PHFs spontaneously aggregate into neurofibrillary tangles (NFTs).

Many scientists postulated that abnormal and excessive phosphorylation precedes tau aggregation and these aggregates are believed to be the toxic species in tauopathies. However, some experimental evidence suggests that filamentous inclusions of tau may not be responsible for neuronal dysfunctions [214–216]. Cowan and co-authors [217,218] have shown that highly phosphorylated wild-type human tau causes behavioral deficits resulting from synaptic dysfunction, axonal transport disruption, and cytoskeletal destabilization *in vivo* in the absence of neuronal death or filament/tangle formation. Physiological and pathological tau species include: monomers, dimers/trimers, small soluble oligomers, insoluble granular tau oligomers, filaments, pretangles, large non-fibrillar tau aggregates, neurofibrillary tangles and ghost tangles [219]. There is a body of evidence, which is still not broadly accepted, that among of all these tau species small soluble tau oligomers are the most toxic and filamentous and fibrillar tau is neither necessary nor sufficient for tau-induced toxicity, and may even represent a neuroprotective strategy [218–221]. Tau dimers and oligomers are considered to be intermediates between soluble tau monomers and insoluble tau filaments. The data suggest that dimers and trimers of tau can suppress axonal transport and cause significantly greater loss of synapses and neurons resulting in stronger memory deficits than tau monomers and fibrils [222,223]. Berger *et al.* [224] in rTg4510 mouse model and Sahara *et al.* [225] in human AD brains have shown that tau 140-kDa dimers appeared at very early stages of disease when memory deficits were evident in the absence of tangle formation. It has been suggested that formation of NFTs is a protective response that ultimately fails [226] (Figure 2).

### 6.2. Tau and Microtubule Instability

The interaction between tau and microtubules is greatly decreased by tau phosphorylation at Ser262 and Ser356 [85]. Other phosphorylation sites shown to have some effects on microtubule association are Ser205, Ser212, Ser214, Thr231, Ser235, Ser396 and Ser404 [227–229]. However, the mechanism leading normal tau to become overphosphorylated and disengaged from microtubules to form tau inclusions remains unclear. Some scientists postulated that in this process reversible lysine acetylation is engaged [68,230,231]. Since acetylation neutralizes charges in the microtubule-binding domain, aberrant acetylation may interfere with the binding of tau to microtubule leading to tau dysfunction [230]. Lys280, in the region ^275^VQINKKLDLS^285^, is one of three lysine residues most critical in modulating tau-microtubules interactions. Increase tau acetylation on Lys280, impairs the interaction with microtubules and increases the pools of cytosolic tau available for pathological aggregation [68].

Although most data on microtubule assembly and pathological tau have been obtained using PHF-tau from AD patients, there is agreement that PHF-tau proteins fail to bind with microtubules [232,233]. Abnormally phosphorylated tau isolated from brain homogenates of AD patients (AD p-tau) comprises little overall activity, but dephosphorylation with alkaline phosphatase recovers its normal activity to a level similar to acid-soluble tau. Microtubule assembly is inhibited in the presence of AD p-tau while tau-tau interactions are facilitated. These studies implicate abnormal phosphorylation of tau in the breakdown of microtubules in affected neurons in AD not only because the altered protein has little microtubule-promoting activity but also because it interacts with normal tau, thereby reducing the amount of “healthy” tau even further. The collapse of microtubules is an important event of neurofibrillary degeneration induced by the aggregation of tau proteins in nerve cells. Findings of the last studies show that interactions between tau and microtubules are more complex than they thought. Some data [51] provide evidence that microtubules promote tau oligomerization on their surface. Additionally Duncan and Goodson [234] have found that microtubules induce rapid formation of tau filaments *in vitro* and that this process probably does not require phosphorylation of tau. It is a question if tau filaments assembly by microtubules might play a role in the formation of Alzheimer’s-associated PHF or NFTs *in vivo*.

A corollary of the abnormalities in tau-microtubule interactions is the progressive break-down of the cytoskeleton, synaptic withdrawal [235,236], and after a brief period of survival, neuronal death and subsequent dementia [237,238]. The cell will experience lysis so that tau is liberated into the extracellular space. Here, tau has high affinity to molecules like sulfated glycosaminoglycans (sGAG), which further promote its polymerization, and upon glycation of the polymers stabilize tau into extracellular neurofibrillary tangles [239].

### 6.3. Tau and Neuronal Transport Defects

In postmitotic neuronal cells, one likely tau/microtubule-dependent function whose abnormality could easily lead to neuronal cell death is axonal transport [240,241]. Indeed, several neurodegenerative disorders are linked to disturbances in cellular cytoskeleton which controls polarized cargo trafficking pathways in neurons [242–244].

The microtubule and F-actin cytoskeleton might act as specific transport roads for intraneuronal trafficking. In the axon and dendrites, transport occurs bidirectionally, from the cell body to the periphery (anterograde transport) and from the periphery to the cell body (retrograde transport). These different directions of transport depend on the polarity of the cytoskeletal tracks. Microtubules are the polar structures: in the axon and the distal dendrites, the plus end (the fast growing end) points distally, whereas in the proximal dendrites, the polarity is mixed [245].

Motor proteins are responsible for the intracellular transport of a wide variety of components and for positioning them along the axon with high spatial-temporal precision. Three different classes of motors are involved in this task: dynein and kinesin, which transport cargoes toward the minus and plus ends of microtubules, respectively, and myosin, responsible for the transport along actin filaments [246–248]. Members of the kinesin superfamily of proteins (KIFs) [249] are known to drive anterograde axonal transport. Cytoplasmic dynein is the major minus end-directed microtubule motor in the neuron and is involved in retrograde axonal transport [250].

The tau, both as microtubule stabilizing and scaffolding protein could be involved in intraneuronal transport. Neurons containing the polar PHFs exhibit severely impaired anterograde transport along axons as well as basal dendrites; transport in apical dendrites is also impaired but in a retrograde-specific manner [251]. New insight into the role of axonal transport in neurodegenerative diseases stems from the observation that proteins accumulated in AD brains can modulate kinesin-1 receptors [252,253]. Overexpression and mislocation of tau proteins appear to modulate kinesin-1 based transport [147,241] by direct inhibition of motors on microtubule tracts, and this can lead to transport disruption for numerous cargoes, including APP vesicles, mitochondria, and peroxisomes, which could explain the energy deprivation and the oxidative stress sensitivity of AD neurons [249,254]. Disturbance of anterograde transport of microtubules slows down exocytosis and affects the distribution of mitochondria which become clustered near to microtubule organizing center (MTOC). The absence of mitochondria and endoplasmic reticulum in the peripheral regions of axons cause a decrease in glucose and lipid metabolism and ATP synthesis and loss of calcium homeostasis [16,255] that leads to a distal degeneration process.

### 6.4. Tau and Neurotrophin Signaling

Since tau controls the bidirectionality of axonal motor-driven transport in a concentration-dependent manner and differentially modulates the kinesin and dynein activity along microtubule tracks [12], defective intracellular trafficking of cargoes, including neurotrophins, could be due to an increased expression level of this protein [256–258] or to its altered intracellular localization [259] or excessive phosphorylation [231,260]. To this regard, the finding that the retrograde transport of I-125-NGF and activated TrkA receptors is inhibited by colchicine, a drug that interferes with the polymerization of microtubules [261,262], suggests that an altered function of tau protein may account for age-related deficiency of long-range neurotrophin signaling in cholinergic neurons. There is good evidence that the retrograde axonal transport of the active NGF-p-TrkA complex involves dynein [263–266], because inhibition of the dynein ATPase activity reduces the retrograde axonal transport of exogenous ^125^I-labelled NGF in sympathetic and sensory neurons *in vivo* [267]. Moreover, TrkA can directly associate with the juxtaposed membrane domain of dynein light chains [265] and phosphorylated TrkA in vesicles can attach and be transported within dynein motors [263]. One candidate protein that is implicated in TrkA transport is a light chain of the dynein motor complex, Tctex-1. Co-immunoprecipitation experiments from brain lysates demonstrated that TrkA, Tctex-1, and dynein form a protein complex [268]. Functional dynein-microtubule network is necessary for TrkA signaling to intracellular Rap1 and MAPK1/2 [269].

The hypothesis that the failure of tau-mediated axonal transport might be responsible for the lack of trophic support in aged or AD brains [59,60,270,271] is supported by several findings. In our study [59], we injected fluorogold (FG) into neocortex and hippocampus of young adult and 24 month old rats and confirmed that the number of retrogradely labeled FG positive neurons was significantly lower in subdivisions of the basal forebrain of aged rats. At the same time, tau immunostaining was restricted to neurites in neurons of the septo-hippocampal projection in young rats, but displayed a mainly somatodendritic distribution in aged rats. This redistribution of tau was confirmed by other immunohistochemical markers against p-TrkA, beta-NGF, p-Tau404 and p-Tau231, and GSK-3β, which can phosphorylate serine 404 and threonine 231 [60]. Apart from an overall lower intensity of p-TrkA immunostaining in cortex and hippocampus of aged rats, immunoreactivity for all proteins was high and localized to the soma in old, and to the axonal and at a somewhat lower intensity to the dendritic compartment in young animals. Our data reveal that during aging expression of GSK-3β and three tau protein substrates are reduced in axons and this may severely compromise the efficiency of retrograde cytoskeletal transport.

Lazarov *et al.* [272] report that the anterograde fast axonal transport (FAT) of APP and Trk receptors is impaired in the sciatic nerves of transgenic mice expressing two independent familial Alzheimer’s disease-linked PS1 mutations. Furthermore, familial Alzheimer’s disease-linked PS1 mice exhibit a significant increase in GSK-3β-mediated phosphorylation of the cytoskeletal proteins tau and neurofilaments in the spinal cord, which correlate with motor neuron functional deficits. It was also shown [273] that the loss of the *N*-terminal 25 amino acids of tau, which probably affects its interaction with dynactin/dynein motor complex [274], occurs in cellular and animal models of AD-like neurodegeneration induced by NGF signaling interruption. A crucial role of tau modifications in NGF-dependent neuronal survival was reported by Amadoro *et al.* [168]. They found that an early, transient and site-specific GSK-3β-mediated tau overphosphorylation (3–6 h after NGF withdrawal) at two AD-relevant pathological epitopes (Ser262 and Thr231) is temporally and causally related with an activation of the endogenous amyloidogenic pathway in NGF-deprived hippocampal primary neurons [275].

## 7. Conclusions and Perspectives

Comprehensive investigations have revealed a role for tau protein in the neuronal cytoskeletal collapse in aging and neurodegenerative tauopathies. Highly phosphorylated tau detaches from microtubules and becomes retrogradely transported to the soma where it accumulates as aggregates of tau and ultimately neurofibrillary tangles. Control of tau phosphorylation by inhibiting tau kinases seems a feasible strategy to prevent tau aggregation and its associated pathological effects. Tau excessive phosphorylation appears to be required, but is not sufficient alone, to induce tau aggregation, other tau post-translational modifications are certainly required. However, tau protein regardless of its post-translational modifications, can also be toxic *per se*, and the suppression of tau protein blocks Aβ-induced toxicity and reduces memory deficit. Such data suggest that reduction of the overall tau levels may constitute a neuroprotective strategy to prevent tauopathies. Therefore, studying tau regulation at the transcriptional and translational levels is of great interest in further understanding of the physiological role of tau and its involvement in human pathologies.

Depletion of axonal tau protein will compromise active transport processes and, as exemplified for cholinergic neurons, impinge on the trophic support mechanism involving NGF and its receptors. It is worth noting that collapse of the cytoskeleton may have consequences for a number of processes. These may include axonal and dendritic transport systems, affecting the distribution of proteins, signaling molecules and organelles throughout the cell. Maintaining neuronal shape and contacts with neighboring cells through synaptic afferents and efferents will also be affected. Deterioration of these processes leads to neurodegeneration, neuronal cell death and cognitive impairment. Therefore, prevention of tau dysfunction and maintenance of the neuronal cytoskeleton may provide important therapeutic strategies for the treatment of AD and other tauopathies.

## Abbreviations

17-AAG: 17-*N*-allyamino-17-demethoxygeldanamycin
ABP: actin-binding proteins
AD: Alzheimer’s disease
AMPAR: α-amino-3-hydroxy-5-methyl-4-isoxazolepropionic acid receptor
AMPK: adenosine-monophosphate-activated protein kinase
APP: amyloid precursor protein
a-SN: α-synuclein
ATP: adenosine triphosphate
Aβ: amyloid beta
BDNF: brain-derived neurotrophic factor
CacyBP/SIP: calcyclin-binding protein/siah-1-interacting protein
cAMP: cyclic adenosine monophosphate
CBD: corticobasal degeneration
Cdk5: cyclin-dependent kinase 5
CHIP: chaperone-associated ubiquitin ligase CHIP
CK1: casein kinase 1
CK1: casein kinase 1
DAPK: death-associated protein kinase
DRG: dorsal root ganglia
DS: Down syndrome
Dyrk1A: dual specificity tyrosine-phosphorylation-regulated kinase 1A
E-10: exon 10
E-2: exon 2
E-3: exon 3
EGF: epidermal growth factor
EHD: Eps15 homology domain
ERK1/2: kinase extracellular signal-regulated kinase 1/2
F-actin: actin filaments
FKBB: FK506-binding protein
FTDP-17: frontotemporal dementia with parkinsonism linked to chromosome 17
Fyn: Proto-oncogene tyrosine-protein kinase Fyn
GABA: gamma-aminobutyric acid
G-actin: globular actin monomers
GCs: growth cones
Grb2: growth factor receptor-bound protein 2
GSK-3: glycogen synthase kinase-3
GTP: guanosine triphosphate
HAP1: Huntingtin-associated protein 1
Hsc70: heat shock cognate 70 protein
Hsp70: heat shock protein 70
Hsp90: Heat shock protein 90
Hsps: heat shock proteins
I2: SET/inhibitor 2
JNK: c-Jun amino-terminal kinase
KHC/KIF5: kinesin-1
KIF1: kinesin-3
KIF3: kinesin-2
KIFs: kinesin proteins
KO: knockout
Kv: voltage-gated potassium channels
M1: muscarinic receptor 1
M3: muscarinic receptor 3
MAP2: microtubule associated protein
MAPKs: mitogen-activated protein kinases
MAPs: microtubule-associated-proteins
MAPT: microtubule-associated protein tau
MARKs: microtubule-affinity regulating kinases
MBD: microtubule binding domain
MF: microfilaments
MPTP: 1-methyl-4-phenyl-1,2,3,6-tetrahydropyridine
mRNA: messenger ribonucleic acid
mRNP: messenger ribonucleoprotein
MT: microtubules
MTOC: microtubule organizing center
NF: neurofilaments
NFH: neurofilament heavy
NFM: neurofilament medium
NFTs: neurofibrillary tangles
NGF: nerve growth factor
NHL: neurofilament light
NMDAR: *N*-methyl-d-aspartate receptor
NMR: spectroscopy nuclear magnetic resonance spectroscopy
non-PDPK: non-proline-directed protein kinase
N-WASP: neural Wiskott-Aldrich syndrome protein
PACSIN1/SYNDAPIN1: protein kinase C and casein kinase substrate in neurons protein 1
P-AMPK: activated/phosphorylated AMPK
PCH: Pombe Cdc15 homology proteins
PD: Parkinson’s disease
PDC: parkinsonism dementia complex of Guam
PDPK: proline-directed protein kinases
PHFs: paired helical filaments
PiD: Pick’s disease
Pin1: peptidyl-prolyl cis-trans isomerase 1
PIP3: phosphatidylinositol (3,4,5)-trisphosphate
p-JNK: phospho-c-Jun amino-terminal kinase
PKA: protein kinase A
PP1: protein phosphatase 1
PP2A: protein phosphatase 2A
PP2B: calcineurin
PP5: protein phosphatase 5
PPI: peptidyl-prolyl *cis*/*trans* isomerase
PPs: protein phosphatases
Pre-mRNA: precursor mRNA
PSD-95: postsynaptic density protein 95
PSP: progressive supranuclear palsy
P-tau: phosphorylated tau
Rab5: Ras-related protein Rab-5A
SH3: domain The SRC homology 3 domain
Src/cSrc: proto-oncogene tyrosine-protein kinase Src
TNAP: tissue-nonspecific alkaline phosphatase
TPK: tyrosine protein kinases
TPKI: tau protein kinase I
TPKII: tau protein kinase II
TrkA: neurotrophic tyrosine kinase receptor, type 1
TrkB: neurotrophic tyrosine kinase, receptor, type 2
UPS: ubiquitin proteasome system
